# Trait anxiety modulates fronto-limbic processing of emotional interference in borderline personality disorder

**DOI:** 10.3389/fnhum.2013.00054

**Published:** 2013-03-01

**Authors:** Jana Holtmann, Maike C. Herbort, Torsten Wüstenberg, Joram Soch, Sylvia Richter, Henrik Walter, Stefan Roepke, Björn H. Schott

**Affiliations:** ^1^Department of Psychiatry, Campus Mitte, Charité Universitätsmedizin BerlinBerlin, Germany; ^2^Campus Benjamin Franklin, Charité Universitätsmedizin BerlinBerlin, Germany; ^3^Department of Education and Psychology, Freie Universität BerlinBerlin, Germany; ^4^Department of Behavioral Neurology, Leibniz Institute for NeurobiologyMagdeburg, Germany; ^5^Department of Neurology, Otto von Guericke UniversityMagdeburg, Germany; ^6^Department of Clinical Psychology, University of SalzburgSalzburg, Austria

**Keywords:** borderline personality disorder, cognition-emotion interaction, anxiety, fMRI, amygdala, anterior cingulate cortex

## Abstract

Previous studies of cognitive alterations in borderline personality disorder (BPD) have yielded conflicting results. Given that a core feature of BPD is affective instability, which is characterized by emotional hyperreactivity and deficits in emotion regulation, it seems conceivable that short-lasting emotional distress might exert temporary detrimental effects on cognitive performance. Here we used functional magnetic resonance imaging (fMRI) to investigate how task-irrelevant emotional stimuli (fearful faces) affect performance and fronto-limbic neural activity patterns during attention-demanding cognitive processing in 16 female, unmedicated BPD patients relative to 24 age-matched healthy controls. In a modified flanker task, emotionally negative, socially salient pictures (fearful vs. neutral faces) were presented as distracters in the background. Patients, but not controls, showed an atypical response pattern of the right amygdala with increased activation during emotional interference in the (difficult) incongruent flanker condition, but emotion-related amygdala deactivation in the congruent condition. A direct comparison of the emotional conditions between the two groups revealed that the strongest diagnosis-related differences could be observed in the dorsal and, to a lesser extent, also in the rostral anterior cingulate cortex (dACC, rACC) where patients exhibited an increased neural response to emotional relative to neutral distracters. Moreover, in the incongruent condition, both the dACC and rACC fMRI responses during emotional interference were negatively correlated with trait anxiety in the patients, but not in the healthy controls. As higher trait anxiety was also associated with longer reaction times (RTs) in the BPD patients, we suggest that in BPD patients the ACC might mediate compensatory cognitive processes during emotional interference and that such neurocognitive compensation that can be adversely affected by high levels of anxiety.

## Introduction

Borderline personality disorder (BPD) is a severe mental disorder characterized by behavioral impulsivity, instability in interpersonal relationships, repetitive suicidal behavior, aggression, particularly autoaggressive behavior, and identity disturbance (Lieb et al., 2004; Mauchnik and Schmahl, 2010). Most of these behavioral patterns are assumed to result from affective instability, which in turn might reflect a general emotional hyperreactivity, but also dysfunction in emotion regulation. The ability to regulate negative emotions successfully allows an individual to adaptively respond to stressful experiences, with deficits in emotion regulation often leading to considerable psychological distress (Gross and Muñoz, 1995; Davidson et al., 2000; Gross, 2002; Ochsner and Gross, 2005). Moreover, emotion regulation abilities also affect an individual's social interactions (Lopes et al., 2005). Notably, BPD patients exhibit particularly pronounced deficits in emotion processing in response to aversive interpersonal events, such as perceived rejection, criticism or separation (Stiglmayr et al., 2005; Gunderson and Lyons-Ruth, 2008). On the other hand, the disturbances of social interaction in BPD (Preißler et al., 2010) might also, to some extent, be a consequence of primarily impaired emotion regulation, leading to a vicious circle (Schmahl and Bremner, 2006; Domes et al., 2009). Behaviorally oriented treatments for BPD like Dialectic-Behavioral Therapy (DBT) or Systems Training for Emotional Predictability and Problem Solving (STEPPS) often focus on emotion regulation and its disturbance (e.g., Linehan, 1993; Blum et al., 2008). Therefore, a better understanding of the underlying neural mechanisms might help to further improve therapeutic strategies for this debilitating psychiatric disorder (Brendel et al., 2005; Koenigsberg et al., 2009).

Despite well-documented clinical and experimental evidence for affective instability in BPD, the underlying neural mechanisms are up to now not quite well understood, with previous studies yielding, at least in part, conflicting results (for a recent metaanalysis see Ruocco et al., 2013). Most functional neuroimaging studies of emotional processing in BPD have focused on a fronto-limbic network that includes the amygdala, the anterior cingulate cortex (ACC), the orbitofrontal cortex (OFC), the hippocampus, and the dorsolateral prefrontal cortex (DLPFC). This network is likely to be involved in the processing of social and emotional information, thereby contributing crucially to emotion regulation (Ochsner and Gross, 2005; Phillips et al., 2008). A dysregulation of this network, most prominently in an interpersonal context, is thought to mediate important aspects of the BPD symptomatology (Brendel et al., 2005; Schmahl and Bremner, 2006; Dell'Osso et al., 2010). A recent metaanalysis of studies investigating negative emotion processing suggests that BPD patients exhibit decreased amygdala and subgenual cingulate, but increased insula activity during processing of negative emotions relative to presumably neutral conditions (Ruocco et al., 2013). On the other hand, several studies have reported higher amygdala activation in BPD patients compared to healthy subjects in response to socially relevant negative emotional stimuli, especially fearful facial expressions (Herpertz et al., 2001; Donegan et al., 2003; Minzenberg et al., 2007; Silbersweig et al., 2007; Koenigsberg et al., 2009). In addition to the observed emotional hyperreactivity, studies focusing on cognition-emotion interactions (e.g., emotion regulation tasks, emotional Stroop paradigms or exposure to autobiographical memories) also suggest that dorsolateral and medial prefrontal regions, including the ACC, might exert an inefficient regulatory functioning in BPD patients (Schmahl et al., 2003, 2004; Minzenberg et al., 2007; Wingenfeld et al., 2009). Taken together, these findings point to a weakened inhibitory control of amygdala reactivity by prefrontal cortical structures in BPD patients (Lieb et al., 2004; Lis et al., 2007; Mauchnik and Schmahl, 2010). Studies demonstrating reduced white matter integrity relevant to a fronto-limbic circuitry and altered functional coupling between the amygdala and the OFC (Grant et al., 2007; New et al., 2007; Rusch et al., 2010) have provided further converging evidence for a disturbance fronto-limbic circuitry in BPD. In line with this idea, emotional stimuli have been shown to interfere with cognitive processing in BPD. Patients with BPD exhibit reduced inhibitory control when confronted with aversive information, which is accompanied by reduced mPFC and increased amygdala activation in fMRI (Silbersweig et al., 2007). In addition, the recruitment of prefrontal cortical control mechanisms during emotional Stroop performance is deficient in BPD patients (Wingenfeld et al., 2009).

Several studies suggest that BPD might be inherently associated with more general cognitive deficits that are not specific to emotion processing (Bazanis et al., 2002; Monarch et al., 2004; Ruocco, 2005; Judd, 2012), but might ultimately also result in deficient regulation of negative emotions. Posner et al. for example, reported alterations of an attentional network involved in conflict resolution and cognitive control in BPD patients (Posner et al., 2002). In this case, impaired inhibition and attentional control might constitute the primary mechanisms of impaired emotion regulation and affective instability in BPD. It should be noted, on the other hand, that cognitive performance in BPD patients is highly variable *intra*individually, a phenomenon that has been linked to reduced prefrontal processing efficiency (MacDonald et al., 2006) and, in the case of BPD, might result from the affective instability of the patients (Beblo et al., 2006). This is in line with the notion that inhibitory control in BPD patients is particularly impaired when the irrelevant information to be suppressed is emotionally aversive in nature (Arntz et al., 2000; Korfine and Hooley, 2000; Domes et al., 2006; Sieswerda et al., 2007). It is thus conceivable that alterations of cognitive processing in BPD might rather result from a primary alteration of emotion processing or its regulation, like the well-documented preferential processing of negative emotions in BPD patients (Barnow et al., 2009; Domes et al., 2009; Dyck et al., 2009; Staebler et al., 2009), particularly in interpersonal contexts (Benjamin et al., 1989; Sieswerda et al., 2007). Compatibly, a direct investigation of voluntary emotion regulation in BPD has indeed yielded both increased amygdala activation and decreased recruitment of the OFC in BPD patients relative to healthy controls (Schulze et al., 2011). It seems thus conceivable that cognitive processing in BPD patients is primarily altered under conditions of emotional distress, as the high intensity of the associated affective processes might exhaust the cognitive resources required for successful emotion regulation. In line with this notion, BPD patients have been shown to exhibit an increased amygdala response to faces with negative emotional and even emotionally neutral expressions (Donegan et al., 2003), and despite the fact that multiple negative emotions are found to be elevated in BPD (Jacob et al., 2009; Staebler et al., 2009), amygdala hyperreactivity in BPD patients is most prominently observed in response to fearful faces (Minzenberg et al., 2007). Moreover, BPD patients also exhibit altered mPFC-amygdala connectivity during fear processing (Cullen et al., 2011). On the other hand, self-report measures usually demonstrate elevated trait anxiety in BPD patients, and the individual degree of anxiety also correlates with behavioral measures of reduced inhibition of negative stimuli during cognitive tasks (Domes et al., 2006).

Previous studies demonstrating altered cognitive processing of negative emotional faces have typically used tasks that required an explicit processing of the negative emotional information, such as gender discrimination (Minzenberg et al., 2007) or the emotional Stroop task (Wingenfeld et al., 2009). To better understand how the (inconsistently reported) general alterations of cognitive function in BPD might be brought about, it might be helpful to disentangle the cognitive task at hand from emotional stimuli. In the present study, we used event-related functional magnetic resonance imaging (fMRI) to investigate how incidental, i.e., task-irrelevant emotional interference, might affect behavioral performance and neural mechanisms in an attention-demanding cognitive task in BPD patients. Emotional stimuli have previously been demonstrated to interfere with PFC-dependent cognitive processing in attention-demanding tasks like the Eriksen flanker task (Eriksen and Eriksen, 1974) in the healthy population (Fenske and Eastwood, 2003; Larson et al., 2006; Wiswede et al., 2009; Richter et al., 2011). The presentation of unpleasant pictures from the International Affective Picture System (IAPS) prior to each flanker stimulus has been shown to lead to an increased error related negativity (ERN) compared to trials with neutral or pleasant pictures (Wiswede et al., 2009), and genetically mediated individual differences in aggression and anger have been linked to altered recruitment of the dACC and the OFC in a comparable task using angry vs. neutral faces (Richter et al., 2011). Because emotional reactivity and attentional bias in BPD patients are particularly pronounced during processing of fearful faces (Minzenberg et al., 2007; Jovev et al., 2012) we adapted the modified flanker task with emotional distracters in the background (Richter et al., 2011) to the use of fearful vs. neutral faces as irrelevant background pictures. The effective completion of the task used here required participants to suppress the irrelevant emotional information and focus attention on the relevant cognitive (flanker) task.

Based on current models of BPD and the previously described functional differences in fronto-limbic networks, we expected that BPD patients might exhibit increased amygdala activations to fearful and possibly to neutral faces and reduced DLPFC- and ACC-dependent cognitive control as compared to controls. Specifically, we hypothesized that reduced dACC and DLPFC activation in the patients would be most prominent during incongruent flanker trials with emotional distracter stimuli. Because previous results indicate that trait anxiety might act as a modifier of inhibitory control of emotional information in BPD (Domes et al., 2006), we further hypothesized that neural signatures of emotional interference in the context of fearful vs. neutral distracters might be correlated with individual levels of trait anxiety. To this end, individual differences in anxiety levels were therefore assessed using the State-Trait Anxiety Inventory (STAI, Spielberger and Lushene, 1966), and trait dimensions of anxiety were included as covariates in all analyses and specifically addressed by brain-behavior correlations, in which we aimed to correlate activations of the dACC, a structure presumably involved in cognitive conflict processing, and of the rACC, a brain region supposedly more directly involved in emotion processing, with trait anxiety. In line of their differential role in neurocognitive networks (Margulies et al., 2007), we tentatively hypothesized that dACC activation might correlate negatively with trait anxiety, whereas the rACC might show an inverse pattern.

## Methods

### Participants

Demographic and clinical characteristics of the study groups are presented in Table 1. Subjects gave written informed consent prior to study participation. The study was approved by the ethics committee of the Charité Universitätsmedizin Berlin. Gender differences in neural correlates have been reported for emotion processing (Hamann and Canli, 2004), and gender seems to play an important role in the neurobiology of BPD (Schmahl and Bremner, 2006); therefore only female subjects were included in the study. Participants were all right-handed and between 20 and 46 years old. Borderline patients were recruited at the Department of Psychiatry, Charité Universitätsmedizin Berlin and all met DSM-IV criteria for BPD. All participants were screened with the German version of the Structural Clinical Interview for DSM-IV (SCID-I and II; First et al., 1996, 1997; German version Wittchen et al., 1997), and symptom severity was assessed with the Symptom Checklist (SCL-90-R; Franke, 2002) and the Borderline Symptom List (BSL; Bohus et al., 2001). Diagnosis of BPD was confirmed by a consultant psychiatrist with extensive experience in the diagnosis and treatment of BPD.

**Table 1:** **Demographic and clinical characteristics**.

	***BPD***	***HC***	**Statistics**
Age	25.56 (4.70)	26.83 (5.35)	*z* = −0.596, n.s.
Smoking	yes = 12	yes = 14	*X*^2^_(1)_ = 1.172, n.s.
LPS (sum subtest 3 + 4)	58.13 (11.05)	61.54 (7.10)	*z* = −0.911, n.s.
MWT-B (IQ)	100.25 (12.53)	106.75 (10.32)	*t*_(38)_ = 1.8, n.s.
STAI-trait (trait anxiety; sum)	63.5 (6.70)	32.58 (5.48)	*z* = −5.308, *p* < 0.001
BIS (sum)	79.00 (13.71)	61.92 (8.24)	*t*_(38)_ = −4.82, *p* < 0.001
SCL-90-R (GSI)	1.93 (0.69)	0.29 (0.21)	*z* = −5.304, *p* < 0.001
BSL (sum)	194.68 (59.29)	31.13 (18.55)	*z* = −5.302, *p* < 0.001
BSL: affect regulation (sum)	33.13 (9.34)	4.21 (4.54)	*z* = −5.229, *p* < 0.001
BDI (sum)	28.81 (9.11)	3.96 (2.77)	*t*_(16.87)_ = −10.59, *p* < 0.001

*Mean scores of psychometric measures for the BPD and HC group. Standard deviations are given in parentheses. Statistics: in case of categorical data Chi-square-tests were applied; for continuous data not significant departing from normal distribution independent sample t-tests (t-values reported) were computed; otherwise Mann–Whitney-U-Tests were used (z-values are reported). LPS, Leistungsprüfsystem; MWT-B, Mehrachwahlwortschatztest form B; STAI-trait, State-Trait-Anxiety Inventory II (trait anxiety scale); BIS, Barratt Impulsiveness Scale; SCL-90-R (GSI), Symptom-Checklist (Global Severity Index); BSL, Borderline Symptom List; BDI, Beck Depression Inventory*.

Exclusion criteria were a history of psychotic disorder, major depression at time of participation, current mania or hypomania, a diagnosis of ADHD, and substance dependence within the last six months prior to study participation. Patients had to be free from psychotropic medication for at least 2 weeks prior to participation (6 weeks in case of fluoxetine), and previous use of depot neuroleptics lead to exclusion for at least 6 months. Control subjects should not meet criteria for any current or past Axis I or Axis II disorder (as screened with the SCID I and II). In both patients and healthy controls any neurological disorder and any current medical condition influencing cerebral metabolism (e.g., diabetes, systemic corticosteroid medication) was also considered as an exclusion criterion. One patient was further excluded from further analysis due to below-chance level performance in the (neutral) congruent flanker condition. The final study sample comprised 16 patients diagnosed with BPD and 24 healthy control subjects (HC). The BPD and control samples were carefully matched with respect to age, smoking status, and intelligence as assessed with the “Multiple-Choice Vocabulary Intelligence Test” (“Mehrfachwahl-Wortschatz-Intelligenztest,” MWT-B; Lehrl, 2005) and subtests 3 and 4 of the “Performance Testing System” (“Leistungsprüfsystem,” LPS-3 and LPS-4; Horn, 1983) (see Table 1). Intelligence measures were considered to be a more appropriate measure than mere years of education, as patients often had disruptions of their educational and professional careers resulting from disorder-related periods of prolonged illness and/or hospitalization.

In the BPD group, two patients met the DSM-IV criteria for posttraumatic stress disorder (PTSD) at the time of participation. Further comorbid Axis I psychiatric diagnoses in this sample included the following: past major depression (*n* = 10), substance abuse (*n* = 7), panic disorder (*n* = 1), social phobia (*n* = 1), obsessive–compulsive disorder (*n* = 1), bulimia nervosa (*n* = 2). Comorbid Axis II disorders were: avoidant personality disorder (*n* = 3), dependent personality disorder (*n* = 1), obsessive-compulsive personality disorder (*n* = 1) and histrionic personality disorder (*n* = 1).

Participants completed complementary well-established questionnaires to assess individual differences in psychopathology. Trait anxiety was assessed using the State-Trait-Anxiety Inventory (STAI; Spielberger and Lushene, 1966). We chose to use trait rather than state anxiety as a measure of individual anxiety levels, as BPD patients, due to their affective instability, might show less reliable responses in the STAI-state, and we were also concerned that state anxiety might even show considerable fluctuations in these patients during the course of the experimental session. We further employed the Barratt Impulsiveness Scale (BIS-11; Patton et al., 1995; German version Preuss et al., 2003) to assess impulsivity and the Beck Depression Inventory (BDI II; Hautzinger et al., 1994) to quantify depressive symptoms.

### Experimental paradigm

Participants were scanned while performing a modified version of the Eriksen Flanker task (Eriksen and Eriksen, 1974) with task-irrelevant emotional and neutral distracters (Richter et al., 2011). The flanker stimulus consisted of a central arrowhead, pointing either to the right or left, flanked by four surrounding arrowheads or four dashes on either side. Flanking arrowheads could point either in the same (congruent condition) or opposite direction (incongruent condition) of the central arrowhead. In these conditions, subjects were instructed to respond as fast and accurately as possible to the pointing direction of the target with a button press on the respective side while ignoring the direction of the surrounding arrowheads. Task-irrelevant pictures of neutral or fearful faces were presented in the background of the flanker stimulus (Richter et al., 2011). The experiment consisted of seven experimental conditions, including four primary conditions of interest with the combinations of congruent/incongruent flanker stimuli and emotional/neutral face stimuli. To improve the estimation accuracy of the stimulus-specific BOLD responses, we included a baseline condition, in which the target flanker was surrounded by dashes only, and a blurred face was presented in the background, thus not eliciting a conflict. Furthermore, two stop conditions (congruent and incongruent) were included, in which the response to the target item should be inhibited. Stop trials were included as a behavioral measure of motor impulsivity, but were not considered further in the present analyses and will be reported separately.

**Figure 1:**
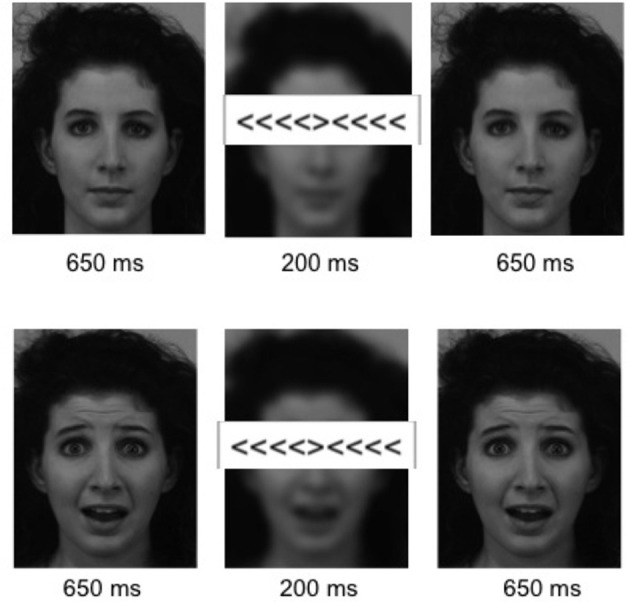
**Stimuli.** Example stimuli for an incongruent flanker condition with a neutral **(Top)** and an emotional **(Bottom)** background pictures. Six hundred and fifty milliseconds presentation of the neutral/fearful face stimulus were followed by 200 ms in which the flanker stimulus appeared at the height of the eyes and the background picture was blurred, ending with another presentation of the face stimulus for 650 ms.

Each trial lasted 1500 ms, beginning with the presentation of a neutral or emotional face stimulus for 650 ms, followed by a 200 ms presentation of the flanker stimulus, during which the face stimulus was blurred, and ending with the respective face stimulus for another 650 ms. Example stimuli and the sequence of one trial are displayed in Figure 1. Flanker stimuli were presented at the location of the face's eyes, thereby requiring subjects to keep the face within the focus of attention. During stop trials a regular flanker stimulus was presented for 100 ms followed by 100 ms of the presentation of a “0” at the site of the target stimulus. The stop conditions were combined with either an emotional or neutral face. Face stimuli were obtained from the Karolinska Directed Emotional Faces database (KDEF; Lundqvist et al., 1998). The experiment lasted approximately 20 min, consisting of 50 trials of each of the emotion x congruency conditions, and 20 emotional and 20 neutral baseline and stop trials respectively, resulting in 280 trials in total. Conditions were presented in random order and response direction (direction of the target stimuli: left/right) was balanced across all conditions. Inter-stimulus intervals were jittered near-exponentially between 2 and 8 s. Stimuli were displayed, and responses were collected using the Presentation software (Neurobehavioral Systems Inc, Albany, CA) and a fiber optic response device (fORP, Current Design Inc, Philadelphia, PA).

### MRI data acquisition

MRI data were acquired on a 3 Tesla Siemens Tim Trio MR tomograph located at the Dahlem Institute for Neuroimaging of Emotion (D.I.N.E.; Cluster Languages of Emotion, Free University of Berlin) with a 12-channel phased array head coil. Because we were interested in both the amygdala and inferior prefrontal structures that typically require opposite tilting of the slice block, we decided to orient the slices in a strict transversal orientation. As displayed **Figure S1**, both the amygdala and the rACC regions-of-interest (ROIs) overlapped in post part with the brain mask, suggesting that signal dropout was negligible.

Functional MRI data were acquired using a gradient, T2^*^-weighted echoplanar imaging pulse sequence (GE-EPI). Thirty-seven adjacent axial slices were acquired along the AC-PC plane in ascending order covering the whole brain, with a 64 × 64 matrix and 192 mm field of view (in-plane voxel size 3 × 3 mm^2^, slice thickness = 3 mm, inter-slice gap = 0.3 mm, *TR* = 2000 ms, *TE* = 30 ms, flip angle = 70°). Structural data were acquired using a 3D T1-weighted MPRAGE sequence (isotropic voxel size 1 × 1 × 1 mm) in a 256 mm field of view (256 × 256 matrix, 176 slices, *TR* = 1900 ms, *TE* = 2.52 ms).

### Data processing and analysis

#### Behavioral data analyses

Behavioral data consisted of mean RTs (for correct responses) and accuracy rates for each subject and were analyzed using SPSS 18 (SPSS Inc, Chicago). These variables were entered into repeated measures analyses of variance (ANOVA), as far as the assumption of normal distribution was met, and subjected to non-parametric test-statistics otherwise. Stop trials were analyzed separately for the dependent variable false alarm rate (failed inhibition of response). The stop trial conditions particularly served the purpose to obtain an additional behavioral measure of impulsivity and were consequently not a factor of interest in the fMRI analyses. All statistical tests employed are listed in Table 2.

Table 2**Mean response times (RT) and accuracy in the four conditions of interest (congruency × emotion) in the Borderline (BPD) and the control group (HC)**.

**Table:** 

**A. Behavior: descriptives**						
	**RT**		**Accuracy**		**FA rate stop trials**	
	***BPD***	***HC***	***BPD***	***HC***	***BPD***	***HC***
**Neutral**					0.213 (0.27)	0.215 (0.27)
Congruent	598.94 (132.25)	665.17 (155.66)	0.961 (0.08)	0.985 (0.03)	–	–
Incongruent	736.69 (160.24)	764.33 (180.83)	0.876 (0.13)	0.949 (0.06)	–	–
**Fearful**					0.259 (0.26)	0.196 (0.24)
Congruent	601.38 (131.07)	670.04 (152.64)	0.977 (0.05)	0.988 (0.03)	–	–
Incongruent	758.31 (166.05)	788.96 (192.73)	0.843 (0.14)	0.949 (0.06)	–	–

**Table:** 

**B. Behavior: statistics**			
**REACTION TIMES**			
**Factor**	***F*_*df*_**	***p***	**Partial Eta squared**
Congruency	81.516_1_	0.000	0.682
Emotion	17.783_1_	0.000	0.319
Group	0.923_1_	0.343	0.024
Congruency^*^emotion	6.190_1_	0.017	0.140
Congruency^*^group	1.819_1_	0.185	0.046
Emotion^*^group	0.183_1_	0.671	0.005
Congruency^*^emotion^*^group	0.001_1_	0.972	0.000

**Table:** 

**ACCURACY**			
**Mann**–**Whitney test**			
	***ME*_cong_**	***ME*_emo_**	***IE*_congemo_**
Mann–Whitney U	147.000	142.500	110.500
Wilcoxon W	283.000	278.500	246.500
Z	−1.245	−1.369	−2.254
R	−0.197	−0.216	−0.356
Exact sig. [2^*^(1-tailed sig.)]	0.222	0.174	0.023

**Table:** 

**Wilcoxon signed ranks test**			
	**(cong-neut + inc-neut)/2 − (cong-emo + inc-emo)/2**	**(inc-neut + inc-emo)/2 − (congneut + cong-emo)/2**	**inc-neut − cong-neut − inc-emo − cong-emo**
Z	−0.873	−4.581	−1.413
R	−0.138	−0.724	−0.065
Asymp. sig. (2-tailed)	0.383	0.000	−0.158

**Table:** 

**FALSE ALARMS**	
**Mann–Whitney test**	
	***ME*_emo_**
Mann–Whitney U	126.000
Wilcoxon W	426.000
Z	−1.860
R	−0.294

**Table:** 

**Wilcoxon signed ranks test**	
	**stop_neut_prop_FA – stop_emot_prop_FA**
Exact sig. [2*(1-tailed sig.)]	0.070
Z	−0.742
R	−0.117
Asymp. sig. (2-tailed)	0.458

*Standard deviations are given in parentheses. Abbreviations: ME_cong_, main effect of congruency; ME_cong_, main effect of emotion; IE_congemo_, interaction effect congruency x emotion*.

#### Fmri data analyses

Image preprocessing and fMRI data analyses were performed using Statistical Parametric Mapping (SPM8, Wellcome Trust Center for Neuroimaging, London, UK; http://www.fil.ion.ucl.ac.uk/spm/software/spm8/) running on Matlab 7.7 (Mathworks Inc., Natick, MA). Data were corrected for acquisition delay and head motion, and subjects' individual T1-weighted MPRAGE images were coregistered to the mean image obtained from motion correction. The MPRAGE image was then segmented using the algorithm implemented in SPM, and EPIs were transformed into the Montreal Neurological Institute (MNI) template space using the normalization parameters obtained from segmentation. Finally, normalized images were smoothed with an isotropic Gaussian kernel of 8 mm full width at half maximum. A temporal high-pass filter with a cut-off frequency of 1/128 Hz was applied to the data to remove low-frequency noise. Serial correlations in time series were removed using an autoregressive model of first order [AR(1)]. For statistical analysis a two-stage mixed effects model was applied. In the first stage, individual general linear models (GLMs) were estimated containing separate covariates for the four conditions of interest [congruent and incongruent flanker condition × fearful and neutral background pictures] and further covariates of no interest for low-level baseline trials, stop trials, error trials, the six rigid-body transformations obtained from motion correction and a single constant representing the mean over scans. Second-level random effects analyses were then computed over the single subjects' contrasts. Only BOLD responses to trials with correct responses were modeled as effects of interest.

In the second stage of the model, single subjects' contrasts of the four conditions were included in two separate within-subject repeated measures ANOVAs for the BPD and the HC group, with the factors subject, flanker (congruent and incongruent), and emotion (fearful and neutral). In the second level analyses, individual differences in anxiety were expected to affect attentional orienting and neural responses to fearful face stimuli, possibly irrespective of diagnosis (Reeck et al., 2012). Similarly, impulsivity has been demonstrated to affect electrophysiological correlates of cognitive monitoring in a flanker task with stop trials in both healthy controls and BPD patients (Ruchsow et al., 2008a,b). As we were interested in both diagnosis-related between-group differences independent of anxiety and impulsivity, but also in the specific influences of trait anxiety, covariates representing individual levels of trait anxiety and impulsivity (obtained from the STAI-trait and BIS questionnaires) were included in all statistical models. Because only two additional factors can be modeled besides the subjects factor in this kind of SPM second level analysis, separate between-subjects ANOVAs were computed for factors group (BPD and HC) and emotion (fearful and neutral); group and congruency (congruent and incongruent) as well as for group and the emotion by congruency interaction [(inc_emo > cong_emo) > (inc_neut > cong_neut)].

Whole-brain voxel-wise comparisons are reported *p* < 0.001, uncorrected, with a minimum cluster size of 10 adjacent voxels. To adjust α-error probabilities for brain regions known to be involved in the paradigm used in this study (Richter et al., 2011), literature-based probabilistic ROIs (Schubert et al., 2008) were generated for all brain regions *a priori* hypothesized, namely the amygdala, the dorsal ACC (dACC), the rostral ACC (rACC), the DLPFC, and the fusiform face area (FFA). The significance level for activation in these ROIs was set at *p* < 0.05, family-wise error (FWE)-corrected for the ROI volumes. Directional t-tests were inclusively masked with the respective F-contrast, thresholded at *p* < 0.05. Correspondence between macroscopic brain anatomy as well as cyto-architectonics and activation foci were determined using a maximum probability map approach (Eickhoff et al., 2006a) as provided by the probabilistic cyto-architectonical brain atlas for SPM (Eickhoff et al., 2005) and areas were labeled according to the publications describing these probabilistic maps (Geyer et al., 1996, 1999; Amunts et al., 1999, 2000, 2005; Morosan et al., 2001; Geyer, 2004; Caspers et al., 2006; Choi et al., 2006; Eickhoff et al., 2006b,c; Malikovic et al., 2007; Rottschy et al., 2007; Scheperjans et al., 2008; Kurth et al., 2010). Literature-based probabilistic ROIs for α-error adjustment were created using a previously described algorithm (Schubert et al., 2008; see Supplementary Information for details).

#### Brain-behavior correlations

For selected core symptoms of BPD the relationship between symptom severity and fMRI activation patterns was investigated by the means of brain-behavior-correlations. Since we used fearful facial expressions as background pictures, the STAI as a measure of trait anxiety was considered to be the most relevant psychometric scale. To avoid circularity in the data analysis (Kriegeskorte et al., 2010), correlations between psychometric data and BOLD-responses were carried out in *a priori* defined ROIs only. Because of their well-characterized role in emotional processing the rACC and amygdala were chosen as ROIs. Further we chose the dACC as a relevant region for contrasts reflecting the interaction of the cognitive process with the fearful face processing. GLM parameter estimates (corrected for the effects of no interest) were extracted from the ROIs for the fearful > neutral contrast (for incongruent and congruent conditions separately) and the incongruent > congruent contrast (for fearful and neutral faces separately) and Pearson's correlations were calculated with the STAI-trait scores in the HC and BPD groups separately. Robustness of correlation values was examined by calculation of Cook's distances (Di), a measure of the influence that single values exert on a correlation (Cook and Weisberg, 1982). In case of single values exceeding an *a priori* defined threshold of Di>4/n (Bollen and Jackman, 1990), the respective subject was excluded and the correlation coefficient recalculated. In order to compare correlation coefficients between groups a bootstrap approach with Monte Carlo approximation was chosen (Efron, 1979). One thousand bootstrap samples of size 16 were generated by independent, random draws with replacement from the original sample and the correlation was calculated for each bootstrap sample. This procedure was applied for the BPD and HC group separately, resulting in 1000 estimates for the correlation coefficient per group and contrast. With the resulting distributions of the correlation coefficients an estimate of the correlation coefficient's standard deviations could be computed. These were used to calculate effect sizes (Cohen's d) for the group differences. Additionally the bootstrap-correlations were entered into Mann–Whitney-U-Tests (BPD vs. HC; all *p*-values were Bonferroni-corrected). Only correlation coefficients significantly differing from zero in at least one of the groups were tested for group differences. *Note:* Brain-behavior correlations were also performed for impulsivity, but those results will be reported separately, together with the stop trial results.

## Results

### Behavior

Descriptive statistics for RTs, accuracy rates and false alarm rates for both groups are presented in Table 2A, and the inferential statistics, including effect sizes are presented in Table 2B.

#### Reaction times

The distribution of RTs did not depart significantly from the predicted normal distribution in either of the conditions (as assessed with the Kolmogorov–Smirnov-Test with Lilliefors significant correction; KS-test; Lilliefors, 1967), neither in the control nor the Borderline group (smallest *p*-value in the KS-test: *p* = 0.11). The ANOVA on RTs yielded a significant main effect of congruency and of emotion [*F*_(1, 38)_ = 81.51, *p* < 0.001 and *F*_(1, 38)_ = 17.78, *p* < 0.001, respectively], as well as a significant congruency by emotion interaction [*F*_(1, 38)_ = 6.19, *p* = 0.017], with RTs being longer in incongruent compared to congruent and emotional compared to neutral trials, yielding their maximum in the incongruent emotional condition. Neither the group main effect [*F*_(1, 38)_ = 0.923, *p* = 0.34] nor the emotion by group, congruency by group nor the three-way interaction reached significance [*F*_(1, 38)_ = 0.183, *p* = 0.671; *F*_(1, 38)_ = 1.82, *p* = 0.185; and *F*_(1, 38)_ = 0.001, *p* = 0.972, respectively]. These results indicate the occurrence of a behavioral conflict effect as well as a differential effect of emotion on the processing of congruent and incongruent flanker stimuli, which did not differ significantly between the BPD and control group.

#### Accuracy

The KS-test on accuracy rates indicated a significant deviation from the normal distribution, thus a non-parametric test procedure was adopted, testing within-subjects effects and between-subjects effects using Wilcoxon-Signed-Ranks-Tests and Mann–Whitney-Tests, respectively. After Bonferroni correction only the main effect of congruency yielded significance (*z* = −4.581, *p* < 0.01).

#### Stop trials

The KS-test on FA rates indicated a significant deviation from the normal distribution, thus a non-parametric test procedure was adopted. Neither the main effect of emotion, nor the main effect of group, nor the emotion by group interaction effect reached significance. This (objective) measure of impulsivity did consequently not indicate any differences in behavioral impulsiveness between the BPD and HC groups.

### Brain responses

Table 3 displays the results of all ROI-based analyses in the dACC, rACC, amygdala, DLPFC, and FFA (*p* < 0.05, small-volume FWE corrected). Tables 4–8 display the results of whole-brain voxel-wise comparisons (*p* < 0.001, uncorrected).

**Table 3:** **Brain activations; ROI-based analyses**.

**Roi, hemisphere**			**Within subject comparisons**				**Between subject comparisons**		
		**Group**	**e > n**	**n > e**	**i > c**	**inter**	**emo**	**cong**	**inter**
dACC (bilat.)	L/R	HC	–	–	0, 17, 43 *p* = 0.010^*^	–	BPD > HC −12, 26, 34 *p* = 0.044^*^	–	–
		BPD	–	–	−6, 20, 43 *p* = 0.078	–			
rACC (bilat.)	L/R	HC	–	6, 50, 1 *p* = 0.086	–	–	–	–	–
		BPD	–	–	–	–			
Amygdala	L	HC	−18, −10, −14 *p* = 0.003^**^	–	–	–	–	–	–
		BPD	−21, −1, −14 *p* = 0.021^*^	–	–	–			
	R	HC	–	–	–	–		–	–
		BPD	30, −1, −14 *p* = 0.040^*^	–	–	24, −4, −23 *p* = 0.007^**^			
DLPFC	L	HC	−42, 11, 2 5 *p* < 0.001^*^	–	−45, 5, 28 *p* = 0.006^**^	–	BPD > HC −27, 29, 31 *p* = 0.099	–	–
		BPD	–	–	–	–			
	R	HC	45, 17, 25 *p* = 0.001^**^	24, 32, 34 *p* = 0.042^*^	45, 8, 28 *p* < 0.001^**^	–	–	–	–
		BPD	45, 26, 13 *p* = 0.041	–	–	–			
FFA	L	HC	−42, −52, −17 *p* < 0.001^**^	–	–	–	–	–	–
		BPD	−39, −46, −17 *p* < 0.001^**^	–	–	–			
	R	HC	33, −67, −11 *p* < 0.001^**^	–	–	–	–	–	–
		BPD	39, −61, −14 *p* = 0.054	–	–	–			

Results of the ROI-based analyses. Peak coordinates are reported. dACC, dorsal anterior cingulate cortex; rACC, rostral anterior cingulate cortex; DLPFC, dorsolateral prefrontal cortex; FFA, fusiform face area;

* FWE-correctable at p < 0.05;

** *FWE-correctable at p < 0.01*.

**Table 4:** **Brain responses; fearful > neutral**.

**Brain structure (area %)**	***H***	**Cluster size**	***Z* (peak)**	**MNI coordinates**		
				***x***	***y***	***z***
**HC**						
Lingual gyrus (BA17: 20%)	R	569	5.46^**^	3	−82	−2
Fusiform gyrus (V4v: 70%)			4.98^**^	30	−70	−11
Lingual gyrus (V3v: 60%)			4.56	21	−79	−5
Middle temporal gyrus (V5: 30%)			3.72	57	−67	1
Inferior temporal gyrus			3.67	51	−73	−5
Fusiform gyrus	L	204	4.86^*^	−42	−52	−17
Lingual gyrus (V4: 30%)			4.39	−21	−79	−14
Inferior occipital gyrus			3.82	−39	−67	−11
Inferior frontal gyrus (p. tria. BA45: 40%)	L	168	4.59	−48	23	−2
Inferior frontal gyrus (p. oper. BA44: 30%)			3.14	−45	14	7
Middle occipital gyrus	R	158	4.54	30	−76	22
Middle temporal gyrus (PGp: 40%)			3.88	51	−76	13
Superior occipital gyrus			3.38	27	−64	31
Superior temporal gyrus	R	118	4.81^*^	45	−31	4
Middle temporal gyrus			4.14	57	−52	4
Inferior frontal gyrus (p. tria. BA44: 40%)	L	115	4.88^*^	−42	11	25
Inferior parietal lobule (7A: 50%)	L	110	4.38	−30	−55	49
Angular gyrus			3.24	−36	−55	37
Inferior frontal gyrus (p. tria.)	R	88	4.77^*^	45	17	25
Middle temporal gyrus	L	70	4.19	−48	−46	7
Thalamus (temporal: 49%)	R	36	4.99^*^	3	−13	1
Amygdala (SF: 50%)	L	24	3.89	−18	−10	−14
Amygdala (LB: 10%)	L	18	4.53	−33	2	−26
Middle occipital gyrus	L	14	3.49	−51	−76	−2
Putamen	L	11	3.69	−30	−10	−8
**BPD**						
Inferior temporal gyrus	L	257	4.61	−39	−46	−17
Fusiform gyrus (V4v: 60%)			4.04	−27	−76	−14
Lingual gyrus			3.87	−24	−52	−11
Inferior occipital gyrus			3.83	−45	−73	−11
Lingual gyrus (BA18: 60%)	R	154	4.58	18	−82	−14
Calcarine gyrus (BA17: 60%)	L		3.88	−9	−91	−2
Inferior frontal gyrus/insula	R	30	4.41	45	26	10
Precuneus (7A: 10%)	L	24	3.94	−9	−67	31
Middle occipital gyrus (BA18: 30%)	R	16	3.48	30	−91	16
Precuneus	R	15	3.72	15	−58	25
Precuneus (5M: 40%)	R	11	3.55	6	−46	67

Clusters of activation for >10 contiguous voxels with p < 0.001, uncorrected. Z, z-score of local maximum;

* FWE-correctable at p < 0.05;

** *FWE-correctable at p < 0.01; Cluster size: in voxels; H, Hemisphere; BA, Brodmann area; hOC4v/hOC5v, human occipital cortex 4/5 ventral; V4/V5, visual area 4/5; SPL, superior parietal lobule; 7A, posterior Superior Parietal Cortex; BA7, anterior part; hIP3, human intraparietal area 3; IPC, Inferior Parietal Cortex; PGa, rostral part of BA39 (angular gyrus), extending from the Inferior parietal sulcus to the temporo-occipital junction; Amygdala SF, superficial; CM, centromedial; LB, laterobasal; 5M, medial area of BA5*.

#### Within-group effects: effect of emotion

**Figure 2:**
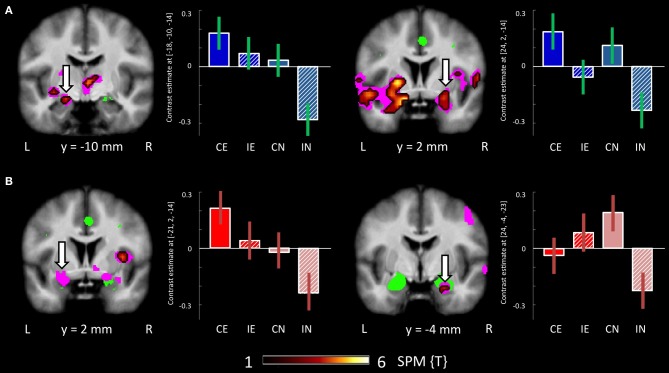
**Brain responses: effect of emotion and congruency in the amygdalae. (A)** Effects in HCs. Left panel: Activation in the left amygdala for the fearful > neutral contrast in the HC group. Right panel: Activation in the right amygdala for the congruent > incongruent contrast in the HC group. **(B)** Effects in BPD patients. Left panel: Activation in the left amygdala for the fearful > neutral contrast in the BPD group. Right panel: Emotion by congruency interaction in the amygdala in BPD patients. Plots depict contrast estimates for the respective peak voxel (±90% confidence intervals). Conditions: CE, congruent emotional; IE, incongruent emotional; CN, congruent neutral; IN, incongruent neutral.

Contrasting the fearful with the neutral condition the control group showed increased BOLD signal in the left amygdala, the inferior frontal gyrus, the middle temporal gyrus, fusiform gyrus, intra-parietal sulcus, and middle occipital gyrus. The BPD group did not show a reliable activation of the left amygdala as well as the fusiform gyrus, lingual gyrus, the inferior frontal gyrus, precuneus and middle and inferior occipital gyri (Tables 3, 4). Emotion-related activation of the FFA survived small-volume correction in the left and right FFA in the HC group (peaks at [−42, −52, −17] and [33, −67, −11]) and in the left FFA in the BPD patients (peak at [−39, −46, −17]). Both groups also showed ROI-correctable activation of the left amygdala during presentation of emotional relative to neutral faces (HC: peak at [−18, −10, −14]; BPD peak at [−21, −1, −14]; see Table 3 and Figures 2A,B, left panel).

In the neutral > fearful faces comparison, healthy controls showed activation increases in the visual cortical and DLPFC structures, as well a trendwise activation in the rACC (Tables 3, 5). The BPD patients, on the other hand, showed an increased activation of the dorsomedial PFC in this contrast.

**Table 5:** **Brain responses; neutral > fearful**.

**Brain structure (area %)**	***H***	**Cluster size**	***Z* (peak)**	**MNI coordinates**		
				***x***	***y***	***z***
**HC**						
Inferior occipital gyrus (BA17: 90%)	R	28	4.96^*^	24	−100	−2
Middle frontal gyrus		16	3.72	24	32	34
Caudate nucleus		12	3.95	9	20	4
**BPD**						
Superior frontal gyrus (BA6: 30%)	R	13	3.91	15	23	61

Clusters of activation for >10 contiguous voxels with p < 0.001, uncorrected. Z, z-score of local maximum;

* FWE-correctable at p < 0.05; ^**^FWE-correctable at p < 0.01; Cluster size: in voxels; H, Hemisphere; BA, Brodmann Area.

#### Within-group effects: effect of congruency

**Figure 3:**
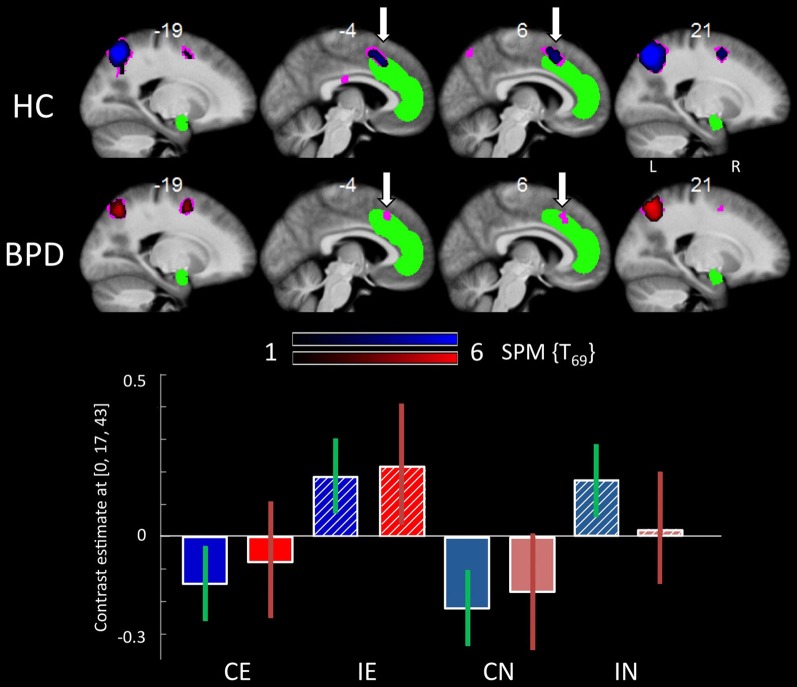
**Brain responses: effects of congruency. Top panel:** Activation in the dACC for the incongruent > congruent contrast in the HC group (upper line) and the BPD group (lower line). **Bottom panel:** Plots depict contrast estimates for the respective dACC ROI analysis peak voxel (±90% confidence intervals) for the HC (in blue) and BPD group (in red) in the four conditions. Abbreviations: CE, congruent emotional; IE, incongruent emotional; CN, congruent neutral; IN, incongruent neutral.

When compared to congruent flanker stimuli, incongruent flanker trials were associated with increased activation in largely overlapping regions in the HC and BPD groups, comprising the inferior and superior parietal lobule, the superior, middle and inferior frontal gyrus, the inferior temporal gyrus, insula, and dACC (Table 6). Corrections for the ROI volumes revealed a significant signal increase in the dACC in healthy controls and a trendwise activation in BPD patients in response to the incongruent flanker stimulus (HC: peak at [0, 17, 43]; BPD: peak at [−6, 20, 43]; see Table 3, Figure 3), whereas activations in the DLPFC were significant after FWE correction in healthy controls only (Table 3). In the congruent > incongruent comparison, both groups showed activation increases in several brain structures (see Supplementary Information: **Table S2** for details). Healthy controls demonstrated greater BOLD signal in both the left and right amygdalae (see Figure 2A, right panel) and the rACC in the congruent condition, whereas BPD patients did not show this activation difference in the amygdala, but only in the rACC (see supplementary **Table S2**). Additionally the BPD group showed a significant activation for the right FFA ROI (Supplementary **Table S2**).

**Table 6:** **Brain responses; incongruent > congruent**.

**Brain structure (area %)**	***H***	**Cluster size**	***Z* (peak)**	**MNI coordinates**		
				***x***	***y***	***z***
**HC**						
Inferior parietal lobule (hIP3:40%)	R	903	6.77^**^	36	−46	49
Superior parietal lobule (SPL/7P: 30%)			6.76^**^	24	−67	52
Supramarginal gyrus (IPC/PFt: 70%)			6.15^**^	48	−31	46
Superior occipital gyrus			5.85^**^	27	−64	34
Angular gyrus (hIP3: 30%)			5.74^**^	30	−58	43
Middle occipital gyrus			3.72	42	−85	10
Superior parietal lobule (SPL/7A: 50%)	L	741	6.72^**^	−21	−64	49
Inferior parietal lobule (hIP2: 40%)			5.65^**^	−42	−37	37
Middle occipital gyrus			5.22^**^	−27	−73	28
Inferior parietal lobule (BA2: 60%)			4.76^*^	−45	−37	52
Inferior frontal gyrus (BA44: 30%)	R	121	5.58^**^	45	5	28
Superior medial gyrus		94	4.02	0	17	43
Superior medial gyrus	L		3.99	−6	14	46
Inferior temporal gyrus	R	63	4.55	57	−55	−11
Precentral gyrus	L	60	4.44	−45	2	31
Superior frontal gyrus	R	55	4.19	24	2	49
Superior frontal gyrus	L	40	3.95	−24	−4	55
Middle frontal gyrus			3.49	−24	5	46
Insula	R	33	4.02	36	20	4
Inferior temporal gyrus	L	33	3.95	−48	−67	−5
**BPD**						
Superior parietal lobule (SPL/7P: 70%)	R	428	5.42^**^	15	−70	55
Superior occipital gyrus			5.03^*^	24	−64	43
Inferior parietal lobule (IPC/PFt: 40%)			4.50	45	−37	49
Middle occipital gyrus			4.40	30	−73	31
Inferior parietal lobule (hIP3: 30%)			4.40	39	−49	49
Middle occipital gyrus (IPC/PGp: 30%)			4.01	39	−79	22
Inferior parietal lobule (hIP1: 40%)	L	138	4.35	−36	−43	40
Inferior parietal lobule (SPL/7PC: 50%)			4.20	−33	−49	49
Superior parietal lobule (SPL/7PC: 60%)			3.99	−33	−52	64
Superior parietal lobule (SPL/7A: 50%)	L	74	5.21^**^	−15	−64	52
Middle frontal gyrus	R	64	4.02	36	2	61
Superior frontal gyrus	L	47	4.07	−21	−1	49
Middle frontal gyrus (BA6: 30%)			3.69	−30	−1	64
Insula	R	46	5.10^**^	33	23	−2
Insula	L	35	4.21	−33	17	1
Inferior frontal gyrus (BA44: 30%)	R	19	3.68	48	8	31

Clusters of activation for >10 contiguous voxels with p < 0.001, uncorrected. Z, z-score of local maximum;

* FWE-correctable at p < 0.05>;

** *FWE-correctable at p < 0.01; Cluster size: in voxels; H, Hemisphere; BA, Brodmann Area; hlP1-3, human intraparietal area 1-3; SMA, supplementary motor area; hOC5, human occipital lobe; V5, visual area 5; 7A,7P, posterior Superior Parietal Cortex, anterior and posterior part of BA7; 7PC, anterior Superior Parietal Cortex; IPC, Inferior Parietal Cortex; Pft, dorsal supramarginal gyrus, rostralmost sector of the IPC*.

#### Within-group effects: interaction congruency-emotion

Testing for the congruency by emotion interaction effect, the corresponding contrast yielded increased activations in the intra-parietal sulcus and the right amygdala in BPD patients. The effect in the right amygdala was robust when correcting for the amygdala ROI volume (Figure 2B, right panel; Table 3). This effect was not found for the HC group. Coordinates and *z*-values are presented in Tables 3, 7.

**Table 7:** **Brain responses; interaction congruency by emotion**.

**Brain structure (area %)**	***H***	**Cluster size**	***Z* (peak)**	**MNI coordinates**		
				***x***	***y***	***z***
**HC**						
Thalamus (Temporal: 20%)		14	3.85	3	−1	1
**BPD**						
Inferior parietal lobule (hIP1: 30%)	R	25	3.94	36	−52	34
Amygdala (LB: 90%)	R	12	3.72	24	−4	−23
Caudate nucleus	L	11	3.71	−15	11	7

*Clusters of activation for >10 contiguous voxels with p < 0.001, uncorrected. Z, z-score of local maximum; Cluster size: in voxels; H, Hemisphere; hIP1, human intraparietal area 1; Amygdala LB, laterobasal*.

#### Between-group effects: group interactions

**Figure 4:**
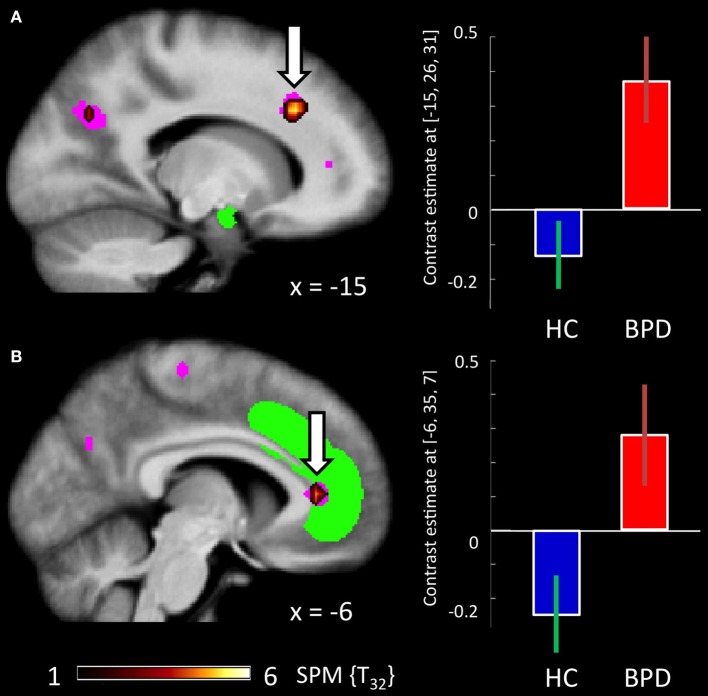
**Brain responses: group by emotion interaction. (A)** BPD_emo_ > neut > HC_emo_ > neut in the dACC. **(B)** BPD_emo_ > neut > HC_emo_ > neut in the rACC. Plots depict contrast estimates for the peak voxel of the respective contrast (±90% confidence intervals) in healthy controls (in blue) and BPD patients (in red).

There were no regions showing higher activation differences in the HC compared to the BPD group as a function of emotion (fearful > neutral), congruency (incongruent > congruent) nor of the congruency by emotion interaction effect. In the fearful > neutral contrast, BPD patients exhibited a higher BOLD signal in the, precuneus, the rACC and in a cluster comprising the dACC and parts of the DLPFC. The elicited activation differences in the dACC were robust after ROI-based FWE correction (peak at [−12, 26, 34]; see Table 3), and the DLPFC cluster showed a trend toward significance when correcting for the respective ROI volume (peak at [−27, 29, 31], FWE-corrected *p* = 0.071; Table 8 and Figure 4). The congruency by group interaction contrast revealed higher signal differences (incongruent > congruent) in the BPD as compared to the HC group in the left pallidum. BPD patients showed higher activation differences for the emotion by congruency interaction effect [(inc-emo > cong-emo) > (inc-neut > cong-neut)] in the temporo-parietal junction (angular gyrus), cuneus, precuneus, middle and superior occipital gyri as compared to healthy controls (Table 8).

**Table 8:** **Brain responses; BPD > HC**.

**Brain structure (area %)**	***H***	**Cluster size**	***Z* (peak)**	**MNI coordinates**		
				***x***	***y***	***z***
**EMOTION**						
Dorsal anterior cingulate cortex	L	26	4.44	−15	26	31
Middle frontal gyrus			3.48	−27	29	31
Precuneus	L	16	3.87	−12	−67	31
Precuneus	R	16	3.70	15	−67	28
Superior frontal gyrus	R	15	3.99	15	35	43
Rostral anterior cingulate cortex	L	11	3.92	−6	35	7
Superior medial gyrus	R	10	4.23	12	62	25
**CONGRUENCY**						
Pallidum	L	18	4.15	−21	2	1
**INTERACTION EMOTION CONGRUENCY**						
Angular gyrus (hIP3: 40%)	R	82	4.15	30	−52	43
Inferior parietal lobule (hIP1: 50%)			3.34	39	−49	34
Middle occipital gyrus			3.24	33	−61	37
Middle occipital gyrus	L	19	4.35	−33	−70	31
Cuneus	R	14	3.87	21	−64	37
Precuneus			3.28	15	−70	40
Superior occipital gyrus	R	14	3.51	21	−76	28
Cuneus			3.51	12	−79	31

*Clusters of activation for >10 contiguous voxels with p < 0.001, uncorrected. Z, z-score of local maximum; Cluster size: in voxels; H, Hemisphere; hIP1/hIP3, human intraparietal area 1/3*.

#### Brain-behavior correlations: effects of trait anxiety

**Figure 5:**
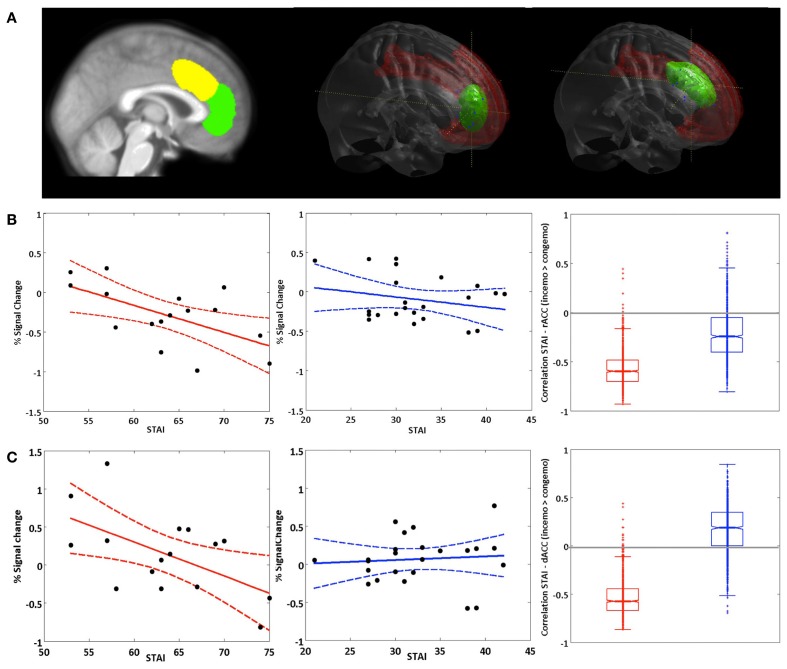
**Brain-behavior correlations: STAI (trait). (A)** Left panel: Non-overlapping ROIs for the dACC (yellow) and rACC (green). Middle and right panel: rendered dACC and rACC ROI. **(B)** Correlation of the STAI trait score with activation in the rACC and **(C)** activation in the dACC in the fearful condition for the contrast inc > cong (solid lines represent regression lines, dashed lines 95% prediction bounds). Left panel: BPD group. Middle panel: HC group. Right panel: Boxplot for the bootstrap-sample correlations (BPD group: red, HC group: blue).

Based on their well-characterized roles in emotion regulation and cognitive control, respectively, we focused our brain-behavior correlations on the rACC and dACC. Pearson correlations of the STAI-trait scores and BOLD responses in the emotional conditions of the congruency effect (incongruent > congruent) yielded significant negative relationships between the two variables in both rACC and dACC ROIs in the BPD group (see Figure 5). Thus, trait anxiety was inversely associated with activation differences between the incongruent and congruent flanker condition when fearful faces were presented as distracters. Notably, these negative correlations were restricted to the patient group, with healthy controls showing no significant relationship between BOLD signal and STAI-trait scores in any of these contrasts or regions. The effect sizes reflecting the group difference in these correlation coefficients were high in both cases (*d* = 1.51 and *d* = 3.71 for the rACC and dACC, respectively) and did differ significantly (*p* < 0.001 for dACC and rACC). Correlation coefficients, bootstrap results and test statistics are given in Table 9 and Figure 5.

**Table 9:** **Brain-behavior correlations; STAI (trait)**.

**Region**	**Contrast**	**Correlation**		**Bootstrap *SD***		**Statistics**	
		***BPD***	***HC***	***BPD***	***HC***	**Mann–Whitney test**	**Cohen's d**
**rACC**							
**Fearful**	Incongruent > congruent	−0.60^*^	−0.24	0.18	0.26	*z* = −30.20, *p* < 0.001	1.62
**Neutral**	Incongruent > congruent	0.31	0.13				
**dACC**							
**Fearful**	Incongruent > congruent	−0.57^*^	0.08	0.19	0.25	*z* = −37.13, *p* < 0.001	3.71
**Neutral**	Incongruent > congruent	−0.28	−0.33				

Pearson correlation coefficients for the BPD and HC group. For the Bootstrap samples Standard deviations of the samples are given. Mann–Whitney tests were calculated for the bootstrap sample (n = 16; N = 1000); Cohen's d was calculated with empirical correlation values (with pooled SD of SD estimates from the bootstrap samples);

* *Significant at p < 0.05*.

In order to assess potential behavioral effects of trait anxiety on performance in the cognitive task, STAI-trait scores were correlated with RT differences of the incongruent fearful and congruent fearful conditions (RT_inc-emo - RT_cong-emo; analogously to the contrast of the BOLD-signal). A positive relationship between trait anxiety and RT differences was observed in both groups (*r* = 0.44 and *r* = 0.19 for BPD and HC, respectively), but reached significance in the BPD group only (*p* = 0.045, one-tailed).

## Discussion

The present study aimed to assess the impact of task-irrelevant emotional information on cognitive processing in patients with BPD. Our results extend previous observations of a dysregulated fronto-limbic circuitry in BPD. By including anxiety and impulsivity as covariates (see “Methods” section for details), we were able to distinguish disorder-related between-group differences and diagnosis-specific correlations of psychopathology and brain activity. Patients showed an interaction between stimulus congruency in the flanker task and emotional interference from the fearful faces in the right amygdala that was not observed in the healthy control group. Furthermore, patients exhibited an emotion-related activation in the rACC/mPFC as well as the dACC that was also absent in controls. Moreover, a disease-specific negative relationship was observed between ACC activity in the emotional incongruent condition and trait anxiety.

### Emotional interference in the flanker task in healthy controls

As evident from the RT and accuracy data, a behavioral conflict effect was elicited by the incongruent trials, and emotional salience of the background pictures showed a more pronounced effect on the processing of incongruent as compared to congruent flanker stimuli. At a neural level, performance of the flanker task was associated with increased activation of the dACC in incongruent relative to congruent trials in the healthy controls, replicating previous results (Botvinick et al., 2004; Fan et al., 2008). Also in line with earlier studies, the amygdala showed higher activation during the presentation of fearful as compared to neutral faces in the HC group (Bush et al., 2000; Whalen et al., 2001; Phan et al., 2004). Results in healthy controls thus confirm the expected effect of the flanker stimuli as well as of the fearful face stimuli, indicating the effectiveness of the current task design.

### Dysregulation of fronto-limbic interactions in BPD

BPD patients, like healthy controls exhibited the behavioral flanker effect with higher error rates and lower RTs in the incongruent condition (Table 2). This was mirrored by fMRI activation of the dACC, the parietal cortex and the dorsolateral and ventrolateral prefrontal cortex in the comparison of incongruent to congruent flanker stimuli, which was also observed in both groups. The dACC is a region consistently found to be activated in tasks involving cognitive or response conflict (Botvinick et al., 2004; Fan et al., 2008). It is believed to play an important role as part of a distributed attention network, with its functions ranging from the modulation of attention and executive functions by influencing sensory systems or response selection, over competition monitoring and error detection to complex motor control (Bush et al., 2000; Botvinick et al., 2004; Mohanty et al., 2007). Activation of the dACC in the BPD patients and HCs during incongruent flanker trials indicates that conflict processing or conflict detection, irrespective of the emotionality of the distracter, does not differ substantially in the patient group. Similarly, both groups showed increased amygdala activation to fearful as compared to neutral faces, also in line with a well-documented responsivity of the amygdala to emotional stimuli, most prominently fearful faces (Costafreda et al., 2008). Therefore, our results do not support the notion that cognitive mechanisms related to attention and conflict processing might be fundamentally altered in BPD patients (Posner et al., 2002). Instead, we observed alterations in more confined subprocesses of emotional interference on cognitive conflict processing.

The amygdala has repeatedly been implicated in the processing of negative emotional states, including fear processing and the recognition of emotional stimuli, especially facial expression of fear (Whalen et al., 2001; Adolphs, 2002; Amaral, 2002; Pessoa et al., 2002; Phan et al., 2002, 2004; Murphy et al., 2003; Fitzgerald et al., 2006; Phelps, 2006). A dysfunction in amygdala reactivity or its regulation in BPD was therefore hypothesized in our study as it might represent an important neural mechanism underlying increased emotional sensitivity and deficient regulation of negative emotions in BPD. In line with this hypothesis we indeed observed differential activation patterns as a function of emotion processing and emotional interference in the bilateral amygdalae. While a significant activation of the left amygdala as a function of emotionality (fearful vs. neutral faces) was found in both groups (Figure 2), healthy controls also showed an increased signal in the left and right amygdala when comparing the congruent with the incongruent flanker condition, irrespective of emotionality. This amygdala activation as a function of congruency was not observed in the BPD patients. This result has to be interpreted with caution due to the lack of a significant effect in the congruency by group interaction, but we tentatively suggest that it might reflect a diminished down-regulation of amygdala activation in the incongruent condition in BPD patients, or, more generally, decreased task-specific modulation of amygdala activity in BPD (Ruocco et al., 2013). On the other hand, the BPD group exhibited a significant interaction of emotion and congruency in the right amygdala, which was not observed in healthy control participants. Previous investigations of amygdala function in the processing of emotional stimuli suggest that the left amygdala is generally recruited more frequently (Costafreda et al., 2008). The right amygdala, on the other hand, appears to be more sensitive to subliminally presented emotional stimuli (Morris et al., 1999; Costafreda et al., 2008), and meta-analyses suggest that, more generally, the left and right amygdalae differ in the temporal dynamics of their responses to emotionally salient stimuli (Sergerie et al., 2008). In the present study, BPD patients exhibited a stronger response of the right amygdala in the emotional incongruent condition as compared to the emotional congruent condition (Figure 2B, right panel). Given the responsivity of the right amygdala to subliminally presented emotional stimuli (Costafreda et al., 2008; Sergerie et al., 2008), we suggest that patients might be able to suppress right amygdala activity by means of emotion regulation in the congruent condition, but not under higher cognitive resource demand of the incongruent condition. An increased responsivity to subliminal negative emotional stimuli in BPD has also been demonstrated in a recent study on attentional bias to fearful faces that was observed in BPD patients during very rapid presentation of the stimuli (Jovev et al., 2012). The notion that the emotion by congruency interaction in the amygdala seen in the patients was not observed in the healthy controls might suggest that, in the healthy population, a right amygdala response, albeit being potentially relatively automatic (Morris et al., 1999), can be suppressed by a demanding cognitive task. In BPD, on the other hand, this suppression of the fast, automatic, right amygdala response might require additional neurocognitive resources and therefore be impaired during performance of demanding tasks. A further aspect of the observed pattern of right amygdala activation in the patient group is the presence of a robust right amygdala response to neutral face stimuli in the congruent condition. One limitation in this context is that participants did not explicitly rate the emotional expressions of the face stimuli. Our finding is, however, compatible with a previously observed negativity bias in BPD patients that is accompanied by an increased amygdala response to neutral facial expressions in BPD (Wagner and Linehan, 1999; Donegan et al., 2003) and with BPD patients showing a heightened emotional sensitivity to facial expressions in general (Lynch et al., 2006).

### The role of the ACC in emotion regulation and the modulatory influence of trait anxiety

The most prominent between-group difference as a function of emotional salience was observed in the dACC and, to a lesser extent, in the rACC/mPFC. BPD patients exhibited somewhat lower dACC activation in the incongruent relative to the congruent flanker condition (albeit not in a direct comparuison with the healthy controls; see Figure 3). On the other hand, an increased dACC—and rACC/mPFC—activation was observed in the patients during presentation of emotional faces (Figure 4), a pattern that showed a trend into the opposite direction in the HC group (Figure 4). Given the comparable behavioral performance in both group, we suggest that this result is indicative of a putatively disorder-specific neural mechanism in BPD patients, leading to an atypical recruitment of an extended ACC region that encompasses both the dACC involved in attentional control and the more rostral region of the pregenual ACC, a portion of the rACC/mPFC complex that has been linked to cognitive processing of emotions, such as the appraisal of fear responses (Mohanty et al., 2007; Etkin et al., 2011).

In addition to the overall increased response of the extended ACC in fearful relative to neutral trials, brain behavior correlations of the STAI-trait scores with both dACC and rACC activation in the emotional high conflict condition (incongruent vs. congruent flanker trials with fearful distracters) revealed a significant negative relationship between trait anxiety and ACC activation during emotional high conflict trials in the BPD, but not in the HC group [*Note:* while the correlation was nominally negative in the HCs as well, it did not approach significance]. Previous studies had demonstrated diminished rACC responses in BPD patients (Minzenberg et al., 2007; Wingenfeld et al., 2009), a finding that could not be confirmed by our study, but instead, our results indicate a disease-specific modulatory effect of trait anxiety on ACC function in BPD. One reason for this apparently diverging result might be the degree of emotion processing elicited by performance of the task at hand in the different studies. In both the gender discrimination task employed by Minzenberg et al. and the emotional Stroop task used by Wingenfeld et al. explicit processing of the emotional information was required for successful task performance. In our study, on the other hand, the face stimuli were completely task-irrelevant, and any attention directed to them could have interfered with performance. We tentatively suggest that patients were largely successful at allocating additional cognitive resources to ACC-dependent emotion regulation and, by upregulating activity of the rACC (and dACC), they were able to compensate for their reduced processing efficiency (possibly similarly to patients with deficits in PFC-dependent cognitive control; see MacDonald et al., 2006) and thus performed the task with a performance largely comparable to that of healthy controls. On the other hand, the patients' ability to recruit ACC regions in situations requiring a higher focus of attention seems thus to be detrimentally affected by their individual degree of trait anxiety. As evident from the brain-behavior correlations, the individual STAI-trait scores were specifically associated with the differential activation in the ACC in the incongruent as compared to the congruent condition with emotional distracters. It thus seems that the impact of higher anxiety on ACC activation in the BPD group only becomes relevant, when the task is sufficiently demanding, and the influence emotional distracters exert over cognitive processing therefore needs to be suppressed. Compatibly, trait anxiety showed a positive correlation with RTs in the BPD group, suggesting that higher anxiety might act as an endogenous attention setting (Reeck et al., 2012) and thereby lead to dysfunctional allocation of cognitive resources to processing of the emotional distracters and adversely affect the ACC-mediated compensatory mechanisms. The observed negative relationship between anxiety and ACC activation is compatible with previous results suggesting a relationship between anxiety and deficient inhibition as well as altered processing of negative information in BPD patients (Domes et al., 2006). While Domes and colleagues observed most pronounced effects of anxiety for state rather than trait anxiety, our results suggest that, at the level or brain activity and subtle RT differences, trait differences of individual anxiety might exert qualitatively similar effects.

While the negative correlation between ACC activation and trait anxiety was restricted to the patient group here, a recent study also reported a similar result in healthy participants (Klumpp et al., 2011). In that study, trait anxiety inversely predicted the response of the rACC to attended relative to unattended angry faces, while no comparable negative correlation was observed for fearful faces. The authors suggested that the attended angry faces might pose a stronger perceived direct threat than the fearful faces. In the present study, faces were always unattended, and no relationship between ACC activation and trait anxiety was observed in the HC group. In BPD patients, on the other hand, the face stimuli were apparently sufficiently salient that the negative relationship of trait anxiety and ACC activity was observed to faces that were not attended and most likely signaled an indirect rather than a direct threat. This observation is compatible with the notion that BPD patients exhibit a cognitive processing bias toward emotionally negative, socially salient stimuli (Barnow et al., 2009; Dyck et al., 2009).

While we had initially hypothesized that trait anxiety might differentially correlate with dACC vs. rACC activation, we observed that the increased activation in the emotional condition irrespective of congruency as well as the negative correlation of the BOLD signal in the emotional incongruent condition with trait anxiety were observed in both the dACC and the rACC. Such an apparently cooperative activation of the dACC, a brain structure that is primarily thought to be involved in cognitive conflict processing, and the pregenual ACC, a region that is thought to belong to a network of regions associated with the regulation of affective processing (Bush et al., 2000; Mohanty et al., 2007; Etkin et al., 2011), may at first appear somewhat counterintuitive, as the two structures are generally thought to belong to distinct networks that are, at least during rest, often found to be negatively correlated (Margulies et al., 2007). However, studies of emotion regulation have shown that dACC activation is commonly found during voluntary, explicit regulatory processes like reappraisal, whereas rACC activation might reflect automatic shifting of attention toward or away from aversive emotional information (Phillips et al., 2008). In the present study, it seems conceivable that participants might have employed a mixed strategy comprising both voluntary and automatic emotion regulation strategies. Moreover, it has recently been suggested that the dissociation of a “cognitive” dACC and an “affective” rACC might no longer be as strongly tenable as previously, with both subregions of the ACC being involved in the regulation of affective processing and in the appraisal of emotional material (Etkin et al., 2011). Specifically, the dACC has been implicated in emotional conflict processing, and activation of the rACC has been linked to appraisal and regulation of emotions, with previous studies having shown diminished rACC responses in BPD patients that were accompanied by increased amygdala activity (Minzenberg et al., 2007).

### Emotional or social interference—or both?

In the present study, when viewing fearful pictures as compared to neutral ones increased activation was observed not only in the amygdala but also fusiform cortex and primary visual processing areas in both groups. Besides modulating emotional responses, the amygdala is thought to interact with sensory processing via backprojections to and a modulation of fusiform cortex and early sensory processing regions (Ledoux, 2000; Vuilleumier et al., 2004; Sabatinelli et al., 2005; Vuilleumier, 2005; Phelps, 2006; Vuilleumier and Pourtois, 2007), thereby enhancing activity in these regions and biasing further perceptual processing through attentional amplification. A subregion of the fusiform cortex has been shown to selectively respond to face stimuli and has thus been commonly referred to as the FFA (Vuilleumier et al., 2004; Vuilleumier, 2005; Vuilleumier and Pourtois, 2007). The observed upregulation of the visual processing stream in response to fearful face stimuli is consistent with the previous literature (Vuilleumier et al., 2001; Sabatinelli et al., 2005) and is indicative of an enhanced representation of fearful as compared to neutral faces in the FFA. In contrast to previous studies (Herpertz et al., 2001; Koenigsberg et al., 2009) we did not find a greater signal increase in the FFA or primary visual areas for BPD as compared to healthy controls. Patients though did show an effect in the FFA with greater signal intensities in the congruent vs. incongruent trials that mirrored the amygdala response pattern observed in the healthy controls. Previous studies suggest that FFA activity often follows the same pattern as that one observed in the amygdala (Vuilleumier et al., 2004; Vuilleumier, 2005). Here, however, Borderline patients exhibited a response pattern to task-irrelevant faces as a function of task difficulty that did not correspond to that of the (right) amygdala, where a complex interaction between congruency and emotional salience of the background pictures was observed. Given the previously reported amygdala response even to neutral faces in BPD (Donegan et al., 2003) and the well-known difficulties in social interactions of BPD patients (Lopes et al., 2005; Koenigsberg et al., 2009; Preißler et al., 2010; Dziobek et al., 2011), we cannot exclude that the response pattern observed here might be specific to face stimuli or possibly social stimuli in general. Future studies should employ other aversive stimuli, such as (non-social) IAPS pictures (Wiswede et al., 2009), to differentiate between effects of social processing and unspecific emotional interference.

### Limitations and directions for future research

The sample size in the present study was modest, though comparable to that of most functional imaging studies of psychiatric populations. Nevertheless a failure to detect possible differences at a behavioral level might be explained by a lack of statistical power, given a complex factorial design like the present one. Also, because our sample consisted of only female patients with relatively typical clinical presentation, we cannot make conclusive inferences for male BPD patients who make up a smaller proportion of all BPD patients and often exhibit atypical clinical features.

A further limitation is that the contribution of comorbid psychiatric disorders in the patient group to the experimental findings remains unclear. However, comorbid disorders are typically observed in the BPD population and exclusion of any comorbidities would have led to the sampling of a non-representative patient group. It should also be noted that the sample did not include any patients with a comorbid generalized anxiety disorder and only one patient with co-morbid panic disorder, making it unlikely that Axis I anxiety disorders can explain the present results.

It must also be note that the present study focused exclusively on fearful faces and anxiety as a negative emotion, but we cannot exclude a different outcome when investigating other negative or positive emotions. While most pronounced emotional interference was to be expected after presentation of fearful faces in BPD patients, future studies should also address the effects of other negative and also on positive emotions on cognitive processing, particularly in the light of a general bias toward negative emotions in BPD. This line of research could also be pursued in other patient groups with affective dysregulation, such as patients with posttraumatic-stress disorder or bipolar disorder.

## Conclusions

In the present functional neuroimaging study, we directly investigated the interference of task-irrelevant emotional information on an attention-demanding cognitive process in BPD. Our results demonstrate that BPD patients exhibit an atypical response of the right amygdala, which might be related to an increased implicit processing of irrelevant negative emotional information. Behaviorally, patients were able to compensate for this, possibly by enhanced recruitment of dACC and rACC structures involved in emotion regulation. The observed disorder-specific negative relationship between trait anxiety and ACC response in the emotional incongruent condition further suggests that anxiety might be an important factor determining the vulnerability of cognitive processing to emotional interference in Borderline patients.

### Conflict of interest statement

The authors declare that the research was conducted in the absence of any commercial or financial relationships that could be construed as a potential conflict of interest.
